# The Role and Regulation of Receptor-Like Kinases in Plant Defense

**Published:** 2007-09-26

**Authors:** Kerry E. Goff, Katrina M. Ramonell

**Affiliations:** Department of Biological Sciences, Box 870344, The University of Alabama, Tuscaloosa, AL 35487-0344 U.S.A

**Keywords:** plant defense, receptor-like kinase, ubiquitin, E3 ligase, disease resistance, elicitors

## Abstract

Receptor-like kinases (RLKs) in plants are a large superfamily of proteins that are structurally similar. RLKs are involved in a diverse array of plant responses including development, growth, hormone perception and the response to pathogens. Current studies have focused attention on plant receptor-like kinases as an important class of sentinels acting in plant defense responses. RLKs have been identified that act in both broad-spectrum, elicitor-initiated defense responses and as dominant resistance (R) genes in race-specific pathogen defense. Most defense-related RLKs are of the leucine-rich repeat (LRR) subclass although new data are highlighting other classes of RLKs as important players in defense responses. As our understanding of RLK structure, activation and signaling has expanded, the role of the ubiquitin/proteasome system in the regulation of these receptors has emerged as a central theme.

## Introduction

Receptor-like kinases (RLKs) consist of a large superfamily of proteins in plants that are structurally similar. They consist of an extracellular region, a single membrane spanning domain and an intracellular kinase domain. Plant RLKs have evolved a diverse complement of extracellular domains (more than 20 have been identified) including leucine-rich repeats (LRR), self-incompatibility (S) domains, epidermal growth factor repeats and lectin domains (Cock et al. 2002; Shiu and Bleeker, 2001). It has been proposed that the diversity of the extracellular domains in plant RLKs reflects their need to evolve rapidly in order to defend against an ever-changing population of ligands produced by pathogens (Shiu and Bleeker, 2001). The ability to recognize both general elicitors and specific pathogens through resistance (R) gene mediated resistance is integral to plant defense. Recent work has highlighted the fact that both elicitor perception in the plant innate immune response and R-gene mediated pathogen specific responses are mediated in many cases by plant RLKs. Most of the RLKs identified as being involved in plant defense are of the LRR-RLK class including the rice Xa21 protein and the Arabidopsis FLS2 and EFR receptors. However, the recent identification in rice of a lysine-motif (LysM) receptor kinase involved in the recognition of the fungal elicitor chitin (Kaku et al. 2006) and a lectin receptor kinase (LecRK) involved disease resistance indicates that other RLK classes may also play important or overlapping roles in plant defense and pathogen recognition.

Studies of RLK structural motifs have given clues to their regulation—particularly by ubiquitylation—and members of the ubiquitin system have recently begun to be implicated as major regulators of plant defense (Zeng et al. 2006). This review will focus on our current understanding of the receptor-like kinases involved in plant defense; describing both RLKs involved in elicitor-mediated or innate immunity and those involved in R-gene-mediated defense. In addition, recent work pointing to the regulation of RLKs, components of their signaling pathways and induced defense proteins by ubiquitylation will be explored.

## RLKs in General Elicitor Recognition

Plants have the ability to recognize invariant pathogen associated molecular patterns (PAMPs) that are characteristic of microbial and fungal organisms but are not found in the host plant. These molecular cues, also termed general elicitors of plant defense, are essential for the pathogen and, upon receptor-mediated perception, they betray the invader to the plant’s surveillance system (Nürnberger et al. 2004). The ability to recognize general elicitors and rapidly initiate defense responses is integral to the basal resistance of plants to most potential pathogens. The identification of receptors involved in the perception of various elicitors and an understanding of their structure and mode of action has been central in shaping our knowledge of elicitor-induced defenses in plant-pathogen interactions (Nürnberger and Kemmerling, 2006).

The first RLK identified involved in elicitor perception was the FLAGELLIN SENSITIVE 2 (FLS2) protein, an RLK with a leucine-rich repeat domain (LRR) that is involved in the perception of flg22, the conserved N-terminal domain of bacterial flagellin (Gómez-Gómez and Boller, 2002). In addition to the extracellular LRR domain, FLS2 has a single membrane spanning domain and a cytoplasmic serine/threonine protein kinase domain. FLS2 appears to play a role in plants similar to that of the TLR5 protein in mammalian systems (Gómez-Gómez and Boller, 2002). Flagellin is a highly conserved PAMP that is recognized by multiple plant species as well as animals. FLS2 was first identified by map-based cloning (Gómez-Gómez and Boller, 2000) and was shown to be expressed in nearly all plant tissues, including flowers, leaves, stems and roots (Gómez-Gómez and Boller, 2002). More recent work using GFP-tagged FLS2 has shown that the receptor is specifically localized to the cell periphery, particularly in the membranes of cells that could serve as a point of entry for pathogens, such as stomatal cells (Robatzek et al. 2006).

Another LRR-RLK that was recently identified as the receptor for a bacterial PAMP is the elongation factor Tu receptor (EFR). EFR is a surface receptor for elf18, a PAMP consisting of the first 18 amino acids of bacterial elongation factor Tu (EF-Tu) a highly conserved protein in bacteria (Zipfel et al. 2006). The EFR receptor belongs to the sub-family LRR-XII and has a domain structure and function similar to that of FLS2. EFR has an extracellular domain comprised of 21 copies of a 24-residue LRR, a transmembrane domain and a cytoplasmic serine/threonine kinase domain. Because the domain structures of EFR and FLS2 were so similar and both elicitors triggered a similar set of responses, Zipfel et al. (2006) investigated whether there was interference or interaction between the two perception systems. When Arabidopsis plants were challenged simultaneously with both flg22 (the conserved portion of bacterial flagellin) and EF-Tu, the plant response was similar to that elicited by flg22 alone, although there was a slight additive effect (Zipfel et al. 2006). Microarray analysis showed a clear correlation between the gene expression patterns in Arabidopsis after treatment with flg22 or EF-Tu. The authors suggest that the lack of differential gene expression between the two treatments may indicate that the plant is recognizing the pathogen-derived elicitor but that the induced response may be generic in nature (Zipfel et al. 2006).

Systemin is an 18 amino acid peptide that acts as an elicitor of defense responses in the Solanaceae and was first isolated in tomato (Ryan and Pearce, 2003). It is released at wound sites in plants (i.e. sites of insect or herbivore feeding) and is perceived by a membrane bound receptor. The systemin cell-surface receptor (SR160) is an LRR-RLK and amino acid analysis of the receptor showed that it contained a signal sequence, a leucine zipper, 25 LRR repeats with a 68 amino acid island between LRR 21 and 22, a transmembrane domain, and a serine/threonine protein kinase domain (Scheer and Ryan, 2002; Boller, 2005). Interestingly, SR160 showed greater than 90% homology to BRASSINOLIDE-INSENSITIVE 1 (BRI1), an Arabidopsis LRR-RLK involved in the perception of the steroid hormone brassinolide (Szekeres, 2003; Boller, 2005). SR160 has been clearly shown to be the tomato BRI1 ortholog (Montoya et al. 2002) but there is debate about whether SR160 and BRI1 are the same receptor in other plant species, particularly in Arabidopsis (Boller, 2005).

Recent work by Huffaker et al. (2006) characterized a new peptide elicitor from Arabidopsis, *At*Pep1. *At*Pep1 is a 23 amino acid endogenous peptide from Arabidopsis leaves that activates the transcription of the defense gene *defensin* (*PDF1.2*) and activates the synthesis of peroxide—both hallmarks of the innate immune response (Huffaker et al. 2006). Constitutive expression of the *At*Pep1 precursor gene *PROPEP1* in transgenic plants was shown to cause constitutive *PDF1.2* expression as well as enhanced resistance to the fungal pathogen *Pythium irregulare* (Huffaker et al. 2006). Interestingly, PROPEP1 orthologs are found in many agricultural species providing new gene targets for the engineering of enhanced disease resistance (Huffaker et al. 2006). In an accompanying article, Yamaguchi et al. (2006) reported the isolation of the AtPep1 receptor from Arabidopsis suspension-cultured cells. The protein, Pep1 receptor (PEPR1), was identified as an LRR-RLK (At1g73080) and was established as the functional receptor through kinetic analysis, photoaffinity labeling, mutant analysis and gain-of-function experiments (Yamaguchi et al. 2006). Further characterization of the ability to activate defense genes and the resistance against pathogens in the null mutants of PEPR1 should yield interesting clues about the response to peptide elicitor signaling. It will be especially useful to compare the signaling pathways initiated between the major classes of elicitors (fungal, bacterial, plant-derived) in order to elucidate the overlap between pathways as well as their unique aspects.

Chitin fragments are well characterized fungal PAMPs and have been shown to produce strong defense responses in plants (Stacey and Shibuya, 1997; Shibuya and Minami, 2001). However, the search for a receptor involved in the perception of chitin has been challenging. Recent work by Kaku et al. (2006) characterized a chitin oligosaccharide elicitor-binding protein (CEBiP) in suspension cultured rice cells. CEBiP was found to be a plasma membrane protein containing two extracellular Lysine motifs (LysM ) that resembled those found in the Nod-factor receptor-like kinases NFR1 and NFR5 involved in nodulation (Radutoiu et al. 2003; Madsen et al. 2003). The CEBiP gene does not have any orthologs in either Arabidopsis or soybean; it seems to be unique to rice. Interestingly, the expression patterns of some genes known to be responsive to chitin was not affected in CEBiP-RNAi cell lines and CEBiP does not contain any intracellular kinase domains that are present in most membrane receptors (Kaku et al. 2006). This strongly suggests that CEBiP requires interaction with another protein and the formation of a receptor complex in order to initiate a successful signaling cascade. A possibility is that chitin recognition in defense responses operates in a similar fashion to the well-documented CLAVATA system involved in meristem development and maintenance. The CLAVATA (CLV) genes (CLV1, CLV2 and CLV3) are responsible for maintenance of meristems through the regulation of the transcription factor WUSCHEL (Diévart and Clark, 2004). In this system, the LRR-RLK CLV 1 interacts with the LRR-RLP CLV2 forming a heterodimer. This heterodimer is then bound by the CLV3 peptide, driving phosphorylation of CLV1 and activating a downstream signaling pathway (Clark, 2001).

## RLKs and R-Gene Mediated Resistance

In addition to elicitor recognition in broad spectrum defense, RLKs also participate in the recognition of pathogen avirulence factors (Avr genes) produced by specific strains of plant pathogens (Lee et al. 2006). The LRR-RLK genes Xa21 and Xa3/Xa26 have been shown to provide resistance against *Xanthomonas oryzae pv. oryza* (*Xoo*) the causative agent of bacterial blight in rice (Song et al. 1995; Sun et al. 2004). The resistance (R) gene Xa21 has been the subject of intense study and is well-characterized. XA21 confers resistance to a broad spectrum of *Xoo* strains (Wang et al. 1996) and acts as a pathogen recognition receptor conferring resistance to strains that produce the AvrXa21 elicitor (Lee et al. 2006). The Xa21 gene codes for an LRR-RLK with 23 copies of a 24 amino acid extracellular LRR domain and an intracellular serine/threonine kinase domain (Song et al. 1995). Xa21’s overall structure is similar to that of the human Toll-like receptors (Kopp and Medzhitov, 2003) which are integral in the mammalian innate immune response to infection and injury (O’Neill, 2002). Interestingly, Xa21 is developmentally controlled; when juvenile rice plants are challenged with *Xoo* they are less resistant than older plants (Century et al. 1999). Xa21-mediated resistance has been shown to increase progressively with plants being susceptible at the juvenile stage and fully resistant at the adult stage (Century et al. 1999).

The *Xa3/Xa26* resistance gene also protects rice against bacterial blight caused by *X*oo (Sun et al. 2004). Recently Xiang et al. (2006) in a series of elegant genetic experiments showed that the R genes *Xa3* and *Xa26* were actually one and the same. Xa3 was fine mapped using a population segregating for only one resistance gene (Xa3) and markers developed from the Xa26 family. The genetic analysis showed that Xa3 co-segregated with the Xa26 gene marker and with markers of other members of the Xa26 family. Additional data including DNA fingerprinting analysis and the correlation of resistance phenotypes and lesion features between rice lines carrying either Xa3 or Xa26 provided further confirmation that Xa3 and Xa26 were separate symbols for one gene (Xiang et al. 2006). Xiang et al. (2006) designated the gene Xa3/Xa26 to indicate the relationship between the two gene symbols. Xa3 and Xa26 were originally mistaken as separate R genes because the gene behaves differently based on the rice variety used and/or the developmental stage of plant being analyzed. These factors both affect Xa3/Xa26 expression and confounded the data (Xiang et al. 2006). Xa3/Xa26 encodes an LRR-RLK with an extracellular domain of 26 imperfect LRRs of 24 amino acids each, a single membrane spanning region and a cytoplasmic serine/threonine kinase domain (Sun et al. 2004). Unlike Xa21, Xa3/Xa26 does not appear to be developmentally regulated, as both juvenile and adult plants exhibit resistance against *Xoo.* The Xa3/Xa26 receptor is constitutively expressed, with highest expression levels found in leaves and weaker expression in sheaths and roots (Sun et al. 2004).

The ERECTA RLK has recently been implicated in plant resistance to bacterial wilt, caused by *Ralstonia solanacearum* (Godiard et al. 2003) and in resistance to the necrotrophic fungal pathogen, *Plectosphaerella cucumerina* (Llorente et al. 2005). The *erecta* mutation was originally characterized for its role in plant organ development and has been extensively studied (Torii et al. 1996). Using quantitative trait loci (QTL) analysis to map the resistance to each pathogen in recombinant inbred lines, both groups (Godiard et al. 2003; Llorente et al. 2005) discovered that the loci conferring the strongest effect corresponded to the *erecta* gene. The ERECTA protein is an LRR-RLK that contains 20 extracellular LRR domains and a cytoplasmic serine/threonine kinase domain (Torii et al. 1996) and resembles the Xa21 resistance gene (Song et al. 1995). Both developmental pathways (i.e. CLAVATA 1 signaling; Diévart and Clark, 2004) and plant defense pathways (FLS2, Xa21) are known to utilize LRR-RLKs in initial signal perception. These data raise intriguing questions about the potential overlap and cross-talk between seemingly dissimilar developmental and defense pathways.

Resistance to Fusarium Oxysporum 1 (RFO1) is an RLK identified in the resistance response to the fungal pathogen *Fusarium oxysporum* forma specialis (f.) *matthioli*. Interestingly, unlike other R genes, RFO1 does not appear to be race-specific and confers broad resistance to pathogenic Fusarium races in Arabidopsis (Diener and Ausubel, 2005). RFO1 encodes an RLK that contains an extracellular wall-associated kinase (WAK) domain, EGF2 and EGF-Ca domains, a transmembrane domain and an intracellular kinase domain. WAK domains have previously been shown to function as receptors and it is an exciting possibility that the WAK domain may be involved in the perception of an as yet unidentified Fusarium-encoded PAMP similar to the perception of Nod factor by Nod-Factor Receptor (NFR) 1 and NFR5 (Diener and Ausubel, 2005). Arabidopsis WAK1 is known to bind a small glycine-rich cell wall protein AtGRP-3 (Park et al. 2001) so it is conceivable that the WAK domain in RFO1 functions in a similar manner.

Recently a B-lectin RLK, termed Pi-d2, was identified as an R gene conferring resistance to the fungal pathogen *Magnaporthe grisea* (*M. grisea*) strain ZB15 (Chen et al. 2006). Pi-d2 is a dominant resistance gene that codes for a RLK in the rice variety Digu. It belongs to the SD-2b RLK subfamily of lectin receptor kinases (LecRKs). The SD-2b subfamily is widely spread throughout rice, but has a limited distribution among other plants (Chen et al. 2006). Pi-d2 contains all of the characteristic regions of a RLK—an extracellular domain, a transmembrane domain, and an intercellular serine/threonine kinase domain—but has additional extracellular lectin domains. Lectin domains have at least two highly conserved regions; one is involved in the binding of monosaccharides and the other consists of a hydrophobic region that forms a conserved hydrophobic pocket. Sequence analysis of Pi-d2 indicates that it lacks parts of the conserved monosaccharide binding region suggesting it most likely cannot bind sugars (Chen et al. 2006). Pi-d2 does contain the conserved hydrophobic pocket region and may utilize this region to bind hydrophobic ligands. These data suggest that Pi-d2 may bind a hydrophobic ligand either released by pathogen hydrolytic enzymes or PAMPs specific to *M. grisea* strain ZB15 released during the infection process (Chen et al. 2006). Northern blots and GFP analysis show that Pi-d2 is expressed constitutively in the plasma membranes of root, stem, and leaf tissues and exposure of rice plants to *M. grisea* strain ZB15 did not induce increased levels of the Pi-d2 RLK (Chen et al. 2006).

## Receptor Kinase Regulation and Ubiquitin—Tag You’re it

**Figure 1:**
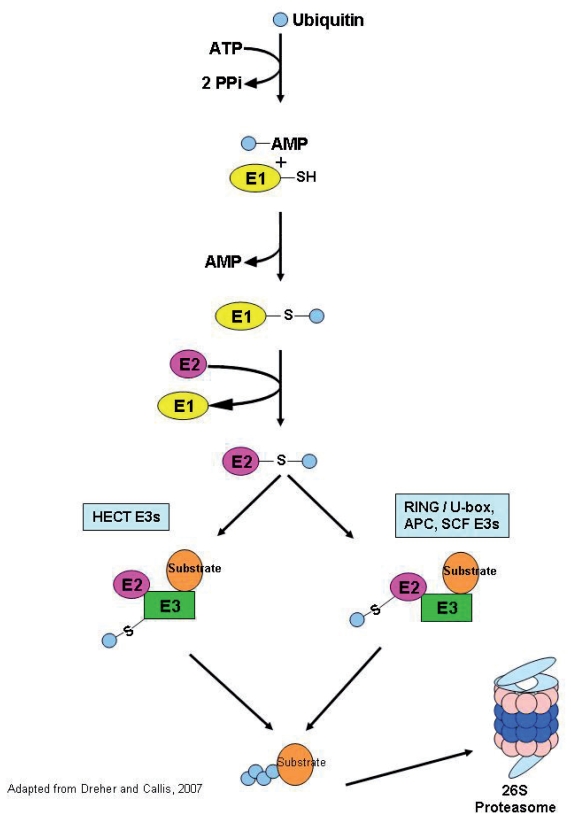
The ubiquitin/26S proteasome pathway. The pathway begins with the activation of ubiquitin by ATP and the subsequent linkage via thioester bond to an E1. This is followed by the transfer of ubiquitin to an E2 enzyme. From here the pathway branches, depending on the type of E3 ligase to which the ubiquitin moiety will be transferred. RING/U-box, APC and SCF-type E3 ligases will facilitate the direct transfer of ubiquitin from the E2 to the target substrate. In the case of HECT-domain E3 ligases, the E2 will first transfer the ubiquitin to the HECT-E3 and then the HECT-E3 will transfer the ubiquitin to the target substrate. In both cases, after a chain of multiple ubiquitin moieties is attached to the target substrate it will be recognized by the 26S proteasome and degraded.

While our knowledge of how pathogens and elicitors are perceived by RLKs is increasing rapidly, less is known about how RLKs and their activated signaling pathways are regulated during the infection process. The most predominant theme that is emerging regarding RLK regulation is that of ubiqitination as a means of targeting receptors for degradation in order to mitigate the plant immune response. In brief, the ubiquitin/proteasome system (UPS) targets proteins for degradation by “tagging” proteins with ubiquitin, a 76 amino acid protein (Fig. 1; Dreher and Callis, 2007). Ubiquitin is attached to proteins by three distinct enzymes: the E1 ubiquitin-activating enzymes, E2 ubiquitin-conjugating enzymes and the E3 ubiquitin ligases. An important and universal feature of the ubiquitin pathway is the hierarchical structure of these three enzymes in eukaryotic genomes (Zeng, 2006). In the Arabidopsis genome there are 2 E1 enzymes, 41 E2s and more than 1200 E3s (Zeng et al. 2006). The diversity of E3 ligases highlights the importance of this enzyme in determining the substrate specificity of the ubiquitin system (Ciechanover A, 1998). There are four families of ubiquitin ligases: HECT (homologous to E6-AP COOH terminus), SCF, APC (anaphase promoting complex) and RING/U-box (really interesting new gene) that are distinguished based on their subunit composition and mechanism of action (Fig. 1; Zeng et al. 2006). Recent experimental work has highlighted the importance of the ubiquitin-proteasome system in plant defense.

Initial structural analysis of the FLS2 protein revealed the presence of a PEST-like motif. PEST regions are rich in the amino acids proline (P), glutamic acid (E), serine (S) and threonine (T) and are typically present in proteins with short intracellular half-lives i.e. <2 hours (Rogers et al. 1986). Mutations of the PEST-like motif in FLS2 lead to a reduction in downstream signaling, suggesting that FLS2 is degraded after interacting with the ligand (Robatzek et al. 2006; Robatzek, 2007). Recent work with GFP-labeled FLS2 has shown that the FLS2-flg22 complex is internalized and degraded after binding to flg22 (Robatzek et al. 2006). Using fluorescent microscopy, Robatzek et al. (2006) captured the movement of vesicles carrying the labeled FLS2 into the cell and away from the plasma membrane. It was also shown that endogenous levels of FLS2 at the membrane were restored within hours. The restoration is a result of de novo synthesis of the receptor rather recycling as experiments showed that the addition of cyclohexamide blocked the return of FLS2 to normal levels. In addition, treatment of FLS2 with a kinase inhibitor blocked the endocytic process. Besides the presence of a PEST domain in FLS2, microarray analysis of flg22 induced genes showed an increase in the overall expression of E3 ligases, further supporting the idea that FLS2 is ubiquitinated and degraded (Robatzek et al. 2006).

The Xa21 RLK was also recently shown to interact with the ubiquitin ligase XB3 (Wang et al. 2006). These experiments by Wang et al. (2006) were the first to show a direct interaction between an E3 ligase, XB3 and an R protein, Xa21. The XB3 protein contains an ankyrin repeat domain that interacts with XA21’s intracellular kinase domain and a RING motif involved in its E3 ligase activity. XB3 acts as a substrate for XA21’s serine/threonine kinase activity and this interaction has been demonstrated *in vitro. In vivo* association between XA21 and XB3 was demonstrated using immunoprecipitation experiments. RNAi studies of XB3 by Wang et al. (2006) showed that in rice lines carrying the RNAiXB3 construct, XA21 accumulated at lower levels in adult plants and resistance of the rice lines to *Xoo* was compromised. However, XB3 accumulates to normal levels in plants with or without the Xa21 gene. These results demonstrate that XB3 stabilizes XA21, is required for the accumulation of the XA21 protein and is necessary for Xa21-mediated resistance. Wang et al (2006) hypothesize that the physical interaction of XB3 and XA21 stabilizes the XA21 protein maintaining its steadystate levels at the plasma membrane. After pathogen infection, the XA21-XB3 complex is required to activate XB3 through transphosphorylation. The activated XB3 protein may then ubiquinate a third protein (a negative regulator) and targets it for degradation (Wang et al. 2006). A second hypothesis is that XB3 self-ubiquitinates, initiating a downstream signaling protein(s). The second hypothesis is supported by work on the interleukin-1 receptor (IL-1) involved in animal innate immunity (Wu and Arron, 2003). Activation of the IL-1 receptor leads to the formation of a receptor/kinase complex that includes the IL-1 receptor, the Toll/IL-1 receptor-domain proteins MyD88 and Tollip and IL-1 receptor associated kinases (IRAKs). The activated IRAKs then dissociate from the receptor complex and associate with the RING-finger ubiquitin ligase TRAF6 (tumor necrosis factor receptor associated factor 6). TRAF6 can then go on to elicit downstream signaling events (Wu and Arron, 2003). The possible similarity between XB3 and TRAF6 functions hints at some exciting parallels between animal and plant defense responses (Wang et al. 2006).

Several other E3 ligases have been identified as involved in plant defense. In response to the elicitor chitin, the RING-finger-type E3 ligases ATL2 and ATL6 in Arabidopsis and EL5 in rice were identified as being rapidly induced (Salinas-Mondragón et al. 1999; Serrano and Guzman, 2004; Takai et al. 2002). The tomato ortholog of Arabidopsis ATL6, LeATL6, has recently been shown to respond to cell wall protein fraction elicitor from the bio-control agent *Pythium oligandrum* and appeared to regulate the jasmonic acid dependent defense related gene expression (Hondo et al. 2007). Our group has identified another RING-finger E3 ligase that is rapidly induced after chitin treatment (Ramonell et al. 2005). Loss-of-function mutations in this gene resulted in enhanced disease susceptibility to the fungal pathogen *Erysiphe cichoracearum*. We hypothesize that the E3 ligase is interacting with a receptor that is involved in chitin recognition but the exact target remains to be discovered (Ramonell et al. 2005). More recently, a RING-finger type protein from pepper CaRFP1 (Capsicum annuum RING-finger protein 1) was identified (Hong et al. 2007). CaRFP1 physically interacts with the basic PR-1 (pathogenicity related-1) protein in leaves of plants infected with both bacterial and fungal pathogens (Hong et al. 2007). Overexpression of CaRFP1 in transgenic Arabidopsis plants conferred disease susceptibility to *Pseudomonas syringae* pv. *tomato* and reduced PR-2 and PR-5 expression. These data suggest that CaRFP1 acts as an E3 ligase that targets PR proteins (Hong et al. 2007).

## Conclusions and Future Directions

Our understanding of plant defense responses, both elicitor-mediated and cultivar-specific R-gene-mediated, has rapidly evolved over the past few years. It is clear that RLKs play an important role in both types of defense responses though only a few RLKs involved in defense have been described. Given the large number of RLKs in plant genomes (>400 in Arabidopsis) it is likely that other RLKs are yet to be discovered that play a role in defense. In both plant and mammalian systems, RLKs are capable of forming heterodimers (Cock et al. 2002; Johnson and Ingram, 2005) though those best characterized in plant defense (FLS2, XA21) are homodimers. It will be interesting to see if RLKs identified in future studies are able to form heterodimers and subsequently stimulate novel defense responses. Another intriguing possibility is that several RLK/ligand complexes, when activated simultaneously, may be able to act in an additive manner initiating specific signaling pathways depending on the combinations of RLKs and ligands present. Currently there is also limited information on the precise components of the signaling pathways activated by RLKs. Further characterization both of the RLKs initiating defense responses and their associated signaling molecules will be essential for a complete understanding of the defense response and how these signaling pathways may interact with one another.

Recent work (Bhat et al. 2005) has shown that lipid rafts or microdomains form in the plasma membrane at points of pathogen entry. In addition, Shahollari et al. (2004) have shown that RLKs containing LRR motifs are concentrated in plasma membrane microdomains and specific signaling proteins are then recruited to these sites. It is possible that RLKs acting in plant defense responses may also be grouped into microdomains upon ligand binding and that particular signaling components associated with a pathogen or elicitor would then be enlisted to begin a unique signal transduction cascade leading to defense. The formation of microdomains would also provide a convenient site for regulatory molecules to efficiently interact with activated RLKs. Consequently, future studies focusing on the formation of microdomains will be significant in deepening our understanding of RLK regulation and in the identification of associated signaling components.

The ubiquitin-proteasome system is emerging as an important regulatory player in plant-pathogen interactions. In particular E3 ligases appear to be key players in the regulation of RLK activity; either by regulating their turnover at the plasma membrane via ubiquitination and targeting them to the proteasome or in the case of XB3 (Wang et al. 2006) by binding at a site in the cytoplasmic region of the RLK thereby stabilizing the receptor at the membrane. In addition to interacting with RLKs in pathogen responses, E3’s also appear to be important factors in the regulation of RLK-induced defense-related proteins (Hong et al. 2007) suggesting several avenues of regulation in the defense response. Since there are over 1200 E3 ligases in the Arabidopsis genome, the possibility that multiple E3 ligases are involved in the response to a single elicitor or avirulence gene is an important question to be addressed. The data of Robatzek et al. (2006) indicate that multiple E3s may be modulating interactions in the RLK-initiated defense response to flagellin suggesting a complex interplay between receptors, their signaling components and regulatory proteins. A priority for future studies will be the determination of which RLKs and E3s specifically interact, identification of their associated signal transduction components, and further insight into the different levels at which the ubiquitin system regulates the response to various pathogens and elicitors.
